# Bis{μ-4-chloro-2-[(2-pyridyleth­yl)imino­meth­yl]phenolato}bis­[chloridocopper(II)]

**DOI:** 10.1107/S1600536808022162

**Published:** 2008-07-19

**Authors:** Jianlan Suo

**Affiliations:** a Department of Chemistry, Baoji University of Arts and Sciences, Baoji, Shaanxi 721007, People’s Republic of China

## Abstract

The title compound, [Cu_2_(C_14_H_12_ClN_2_O)_2_Cl_2_], is a copper(II) dimer where the metal centres are bridged by O atoms from a 5-chloro­salicylaldehyde group. The coordination geometry of each copper(II) centre is distorted square-pyramidal, with two N atoms from a 2-ethyl­amino­pyridine group and two O atoms from a 5-chloro­salicylaldehyde group occupying the basal positions, and with a Cl atom at the apical position. The dimer is centrosymmetric, with a crystallographic inversion centre midway between the two Cu atoms [Cu⋯Cu = 3.103 (9) Å].

## Related literature

For related literature, see: Du *et al.* (2003 ▶); Rojas *et al.* (2004 ▶); Yamada (1999 ▶).
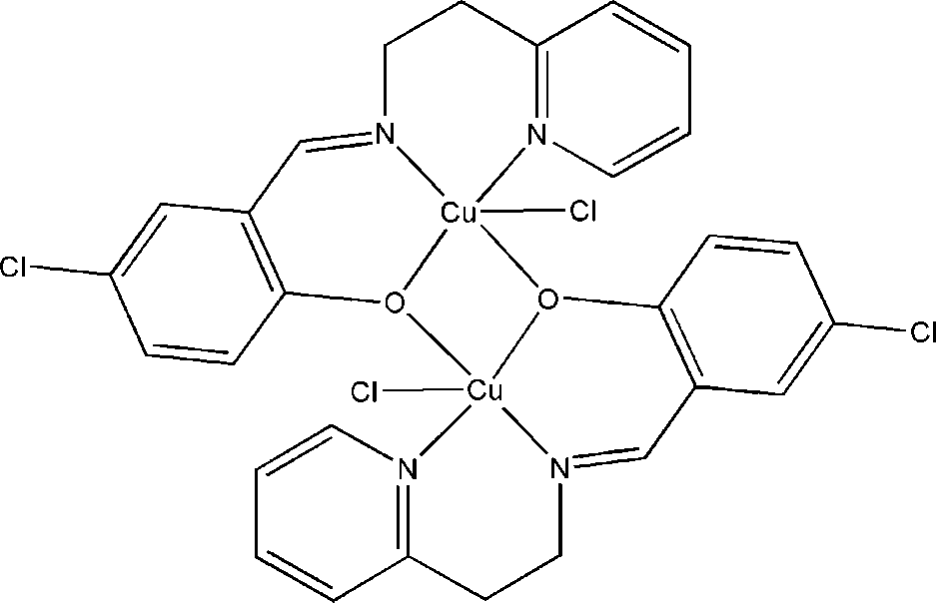


## Experimental

### 

#### Crystal data

[Cu_2_(C_14_H_12_ClN_2_O)_2_Cl_2_]
*M* *_r_* = 717.39
Monoclinic, 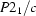

*a* = 9.9703 (10) Å
*b* = 9.0119 (11) Å
*c* = 16.5018 (16) Å
β = 96.0390 (10)°
*V* = 1474.5 (3) Å^3^
*Z* = 2
Mo *K*α radiation
μ = 1.84 mm^−1^
*T* = 298 (2) K
0.50 × 0.42 × 0.02 mm

#### Data collection

Bruker SMART CCD area-detector diffractometer
Absorption correction: multi-scan (*SADABS*; Sheldrick, 1996 ▶) *T*_min_ = 0.460, *T*_max_ = 0.957
7139 measured reflections
2587 independent reflections
2101 reflections with *I* > 2σ(*I*)
*R*_int_ = 0.035

#### Refinement

*R*[*F* ^2^ > 2σ(*F* ^2^)] = 0.031
*wR*(*F* ^2^) = 0.082
*S* = 1.05
2587 reflections
181 parameters
H-atom parameters constrained
Δρ_max_ = 0.38 e Å^−3^
Δρ_min_ = −0.32 e Å^−3^

### 

Data collection: *SMART* (Siemens, 1996 ▶); cell refinement: *SAINT* (Siemens, 1996 ▶); data reduction: *SAINT*; program(s) used to solve structure: *SHELXS97* (Sheldrick, 2008 ▶); program(s) used to refine structure: *SHELXL97* (Sheldrick, 2008 ▶); molecular graphics: *SHELXTL* (Sheldrick, 2008 ▶); software used to prepare material for publication: *SHELXTL*.

## Supplementary Material

Crystal structure: contains datablocks I, global. DOI: 10.1107/S1600536808022162/gw2043sup1.cif

Structure factors: contains datablocks I. DOI: 10.1107/S1600536808022162/gw2043Isup2.hkl

Additional supplementary materials:  crystallographic information; 3D view; checkCIF report

## Figures and Tables

**Table 1:** Selected bond lengths (Å)

Cu1—O1^i^	1.9547 (18)
Cu1—N2	1.958 (2)
Cu1—Cl2	2.3187 (9)

Symmetry code: (i) 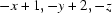.

**Fig. 1.**
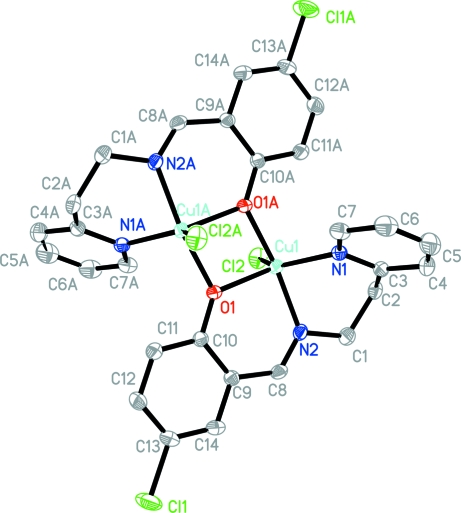
The structure of the title compound in 30% probability ellipsoids. H atoms are omitted for clarity. [Symmetry code: (A)-x + 1, y, -z + 1]

**Fig. 2.**
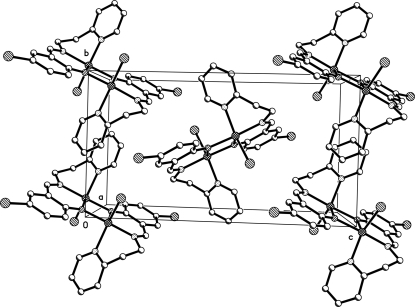
The molecular packing of (I) viewed along the b axis.

## supplementary crystallographic information

### Comment

Transition metal complexes containing Schiff base ligands have been of great interest for many years (Yamada, 1999). These complexes play an important role in the coordination chemistry related to catalysis and enzymatic reactions, magnetism and molecular architectures. The complexes of salicylaldehyde with polyamines and bis(phenoxo) bridged dinuclear copper(II) complexes are rare. As an extension of the work on the structural characterization of Schiff base complexes, the crystal structure of a mononuclear copper(II) compound, (I), is reported here.

The molecular structure of complex (I) is defined by two [CuLCl] units [4-chloro-2-pyridylethylamine-phenolato], which are bridged by two atoms from 5-Chlorosalicylaldehyde, in such a way as to define a central N_2_CuO_2_CuN_2_ core. Additionally, there is an Cl atom from CuCl_2_.2H_2_O completing the pentacoordination of each Cu atom, thus defining a slightly distorted square-based pyramidal coordination for the metal centres. The basal square of the pyramid is defined by two N atoms (N1 and N2) from 2-ethylaminopyridine and two O atoms from 5-Chlorosalicylaldehyde [O1 and O1A; symmetry code: (A)-*x* + 1, *y*, -*z*].

The Cu—Cl2 distance is 2.3187 (9) Å,, which is a rather long value for the normal length of this kind of bond (2.0512 Å). A similar value has been reported for [Cu_2_(/*m*-oxalato) (dipyridylamino)_2_(CH_3_—CN)_2_](ClO_4_)_2_ (Du *et al.*, 2003). The Cu—Cu distance of 3.103 (9) Å is close to this kind of complex (Rojas *et al.*, 2004). Consistently, the O—Cu—O1A angle is 78.44 (8) °. The atom sequence Cu—O1—Cu1A—O1A is a rather parallelogram. The Cu—O1 and Cu—O1A distances are 1.9547 (18) Å and 2.0500 (19) °, respectively.

### Experimental

5-Chlorosalicylaldehyde (0.1 mmol, 15.7 mg), CuCl_2_.2H_2_O (0.1 mmol, 17.05 mg) and 2-ethylaminopyridine(0.1 mmol, 122.2 mg) were dissolved in methanol (10 ml). The mixture was stirred for 30 min at room temperature to give a clear brown solution. After allowing the resulting solution to stand in air for 11 d, brown block-shaped crystals of (I) were formed on slow evaporation of the solvent. The crystals were collected, washed with methanol and dried in a vacuum desiccator using anhydrous CaCl_2_ (yield 54%). Analysis found: C 46.84°, H 3.35° N 7.80°.calculated for Cu_2_(C_14_H_12_N_2_OCl_2_)_2_Cl_2_: C 46.86%, H 3.35%, N 7.81°.

### Refinement

All H atoms were placed in geometrically idealized positions and constrained to ride on their parent atoms with C—H distances in the range 0.93–0.97 Å and *U*_iso_(H) = 1.2*U*_eq_ or 1.5*U*_eq_(C/O)

### Crystal data

**Table:**

[Cu_2_(C_14_H_12_Cl_1_N_2_O)_2_Cl_2_]	*F*_000_ = 724
*M**_r_* = 717.39	*D*_x_ = 1.616 Mg m^−^^3^
Monoclinic, *P*2_1_/*c*	Mo *K*α radiation λ = 0.71073 Å
*a* = 9.9703 (10) Å	Cell parameters from 3383 reflections
*b* = 9.0119 (11) Å	θ = 2.3–28.1º
*c* = 16.5018 (16) Å	µ = 1.84 mm^−^^1^
β = 96.0390 (10)º	*T* = 298 (2) K
*V* = 1474.5 (3) Å^3^	Block, brown
*Z* = 2	0.50 × 0.42 × 0.02 mm

### Data collection

**Table:**

Bruker SMART CCD area-detector diffractometer	2587 independent reflections
Radiation source: fine-focus sealed tube	2101 reflections with *I* > 2σ(*I*)
Monochromator: graphite	*R*_int_ = 0.035
*T* = 298(2) K	θ_max_ = 25.0º
φ and ω scans	θ_min_ = 2.1º
Absorption correction: multi-scan(SADABS; Sheldrick, 1996)	*h* = −11→11
*T*_min_ = 0.460, *T*_max_ = 0.957	*k* = −10→10
7139 measured reflections	*l* = −14→19

### Refinement

**Table:**

Refinement on *F*^2^	Secondary atom site location: difference Fourier map
Least-squares matrix: full	Hydrogen site location: inferred from neighbouring sites
*R*[*F*^2^ > 2σ(*F*^2^)] = 0.031	H-atom parameters constrained
*wR*(*F*^2^) = 0.082	*w* = 1/[σ^2^(*F*_o_^2^) + (0.0359*P*)^2^ + 0.6685*P*] where *P* = (*F*_o_^2^ + 2*F*_c_^2^)/3
*S* = 1.05	(Δ/σ)_max_ = 0.001
2587 reflections	Δρ_max_ = 0.38 e Å^−^^3^
181 parameters	Δρ_min_ = −0.32 e Å^−^^3^
Primary atom site location: structure-invariant direct methods	Extinction correction: none

### Special details

**Table:**

Geometry. All e.s.d.'s (except the e.s.d. in the dihedral angle between two l.s. planes) are estimated using the full covariance matrix. The cell e.s.d.'s are taken into account individually in the estimation of e.s.d.'s in distances, angles and torsion angles; correlations between e.s.d.'s in cell parameters are only used when they are defined by crystal symmetry. An approximate (isotropic) treatment of cell e.s.d.'s is used for estimating e.s.d.'s involving l.s. planes.
Refinement. Refinement of *F*^2^ against ALL reflections. The weighted *R*-factor *wR* and goodness of fit *S* are based on *F*^2^, conventional *R*-factors *R* are based on *F*, with *F* set to zero for negative *F*^2^. The threshold expression of *F*^2^ > σ(*F*^2^) is used only for calculating *R*-factors(gt) *etc*. and is not relevant to the choice of reflections for refinement. *R*-factors based on *F*^2^ are statistically about twice as large as those based on *F*, and *R*- factors based on ALL data will be even larger.

### Fractional atomic coordinates and isotropic or equivalent isotropic displacement parameters (Å^2^)

**Table:**

	*x*	*y*	*z*	*U*_iso_*/*U*_eq_	
Cu1	0.40654 (3)	0.92113 (4)	0.05596 (2)	0.03066 (13)	
Cl1	1.06351 (9)	0.91583 (13)	0.29917 (6)	0.0704 (3)	
Cl2	0.29315 (9)	1.11255 (9)	0.11335 (5)	0.0489 (2)	
N1	0.3451 (2)	0.7177 (2)	0.01064 (14)	0.0326 (5)	
N2	0.4538 (2)	0.8209 (2)	0.16031 (13)	0.0328 (5)	
O1	0.60250 (18)	0.9870 (2)	0.05178 (11)	0.0332 (5)	
C1	0.3444 (3)	0.7399 (4)	0.19511 (18)	0.0451 (8)	
H1A	0.3116	0.7990	0.2380	0.054*	
H1B	0.3796	0.6477	0.2191	0.054*	
C2	0.2278 (3)	0.7060 (3)	0.13033 (19)	0.0431 (8)	
H2A	0.1642	0.6415	0.1538	0.052*	
H2B	0.1814	0.7979	0.1147	0.052*	
C3	0.2706 (3)	0.6337 (3)	0.05563 (18)	0.0378 (7)	
C4	0.2375 (4)	0.4895 (4)	0.0332 (2)	0.0553 (9)	
H4	0.1859	0.4316	0.0648	0.066*	
C5	0.2823 (5)	0.4327 (4)	−0.0368 (3)	0.0684 (12)	
H5	0.2613	0.3357	−0.0526	0.082*	
C6	0.3568 (4)	0.5184 (4)	−0.0824 (2)	0.0557 (10)	
H6	0.3866	0.4812	−0.1300	0.067*	
C7	0.3882 (3)	0.6617 (3)	−0.05733 (18)	0.0423 (8)	
H7	0.4401	0.7205	−0.0882	0.051*	
C8	0.5714 (3)	0.8170 (3)	0.19992 (16)	0.0346 (7)	
H8	0.5789	0.7624	0.2480	0.042*	
C9	0.6936 (3)	0.8864 (3)	0.17933 (16)	0.0304 (6)	
C10	0.7074 (3)	0.9662 (3)	0.10664 (16)	0.0304 (6)	
C11	0.8341 (3)	1.0216 (3)	0.09434 (18)	0.0392 (7)	
H11	0.8453	1.0702	0.0458	0.047*	
C12	0.9435 (3)	1.0062 (4)	0.15249 (19)	0.0429 (8)	
H12	1.0267	1.0463	0.1439	0.051*	
C13	0.9277 (3)	0.9303 (3)	0.22358 (19)	0.0418 (8)	
C14	0.8076 (3)	0.8687 (3)	0.23679 (18)	0.0387 (7)	
H14	0.8005	0.8144	0.2841	0.046*	

### Atomic displacement parameters (Å^2^)

**Table:**

	*U*^11^	*U*^22^	*U*^33^	*U*^12^	*U*^13^	*U*^23^
Cu1	0.0349 (2)	0.0311 (2)	0.02574 (19)	−0.00498 (15)	0.00216 (14)	0.00274 (14)
Cl1	0.0410 (5)	0.1083 (9)	0.0574 (6)	0.0051 (5)	−0.0164 (4)	0.0100 (5)
Cl2	0.0554 (5)	0.0391 (5)	0.0541 (5)	0.0034 (4)	0.0150 (4)	−0.0099 (4)
N1	0.0365 (14)	0.0285 (13)	0.0315 (13)	−0.0010 (11)	−0.0028 (10)	0.0010 (10)
N2	0.0392 (14)	0.0313 (13)	0.0285 (12)	−0.0062 (11)	0.0061 (10)	0.0022 (10)
O1	0.0299 (11)	0.0400 (11)	0.0286 (10)	−0.0046 (9)	−0.0018 (8)	0.0094 (9)
C1	0.048 (2)	0.053 (2)	0.0358 (17)	−0.0115 (16)	0.0077 (14)	0.0078 (15)
C2	0.0405 (18)	0.0408 (18)	0.0485 (19)	−0.0105 (15)	0.0071 (14)	0.0110 (15)
C3	0.0378 (18)	0.0288 (16)	0.0441 (18)	−0.0024 (13)	−0.0078 (14)	0.0052 (13)
C4	0.067 (2)	0.0361 (19)	0.059 (2)	−0.0146 (17)	−0.0094 (18)	0.0057 (17)
C5	0.090 (3)	0.032 (2)	0.077 (3)	−0.001 (2)	−0.022 (2)	−0.0097 (19)
C6	0.073 (3)	0.042 (2)	0.050 (2)	0.0119 (19)	−0.0068 (18)	−0.0130 (17)
C7	0.0445 (19)	0.0434 (19)	0.0376 (17)	0.0064 (15)	−0.0026 (14)	−0.0032 (14)
C8	0.0470 (19)	0.0328 (16)	0.0236 (14)	0.0004 (14)	0.0017 (12)	0.0027 (12)
C9	0.0344 (16)	0.0293 (15)	0.0270 (15)	−0.0006 (12)	0.0009 (12)	−0.0026 (11)
C10	0.0345 (16)	0.0268 (15)	0.0293 (15)	−0.0010 (12)	0.0006 (12)	−0.0015 (11)
C11	0.0358 (18)	0.0423 (18)	0.0393 (17)	−0.0025 (14)	0.0028 (13)	0.0081 (14)
C12	0.0275 (16)	0.0469 (19)	0.053 (2)	−0.0012 (14)	−0.0005 (14)	0.0035 (16)
C13	0.0336 (18)	0.0494 (19)	0.0402 (18)	0.0040 (15)	−0.0070 (13)	−0.0002 (15)
C14	0.0416 (19)	0.0407 (17)	0.0321 (16)	0.0058 (14)	−0.0031 (13)	0.0030 (13)

### Geometric parameters (Å, °)

**Table:**

Cu1—O1^i^	1.9547 (18)	C4—C5	1.381 (5)
Cu1—N2	1.958 (2)	C4—H4	0.9300
Cu1—N1	2.049 (2)	C5—C6	1.353 (6)
Cu1—O1	2.0500 (19)	C5—H5	0.9300
Cu1—Cl2	2.3187 (9)	C6—C7	1.382 (5)
Cl1—C13	1.747 (3)	C6—H6	0.9300
N1—C3	1.339 (4)	C7—H7	0.9300
N1—C7	1.341 (4)	C8—C9	1.441 (4)
N2—C8	1.282 (4)	C8—H8	0.9300
N2—C1	1.478 (4)	C9—C14	1.411 (4)
O1—C10	1.323 (3)	C9—C10	1.417 (4)
O1—Cu1^i^	1.9547 (18)	C10—C11	1.392 (4)
C1—C2	1.525 (4)	C11—C12	1.382 (4)
C1—H1A	0.9700	C11—H11	0.9300
C1—H1B	0.9700	C12—C13	1.381 (4)
C2—C3	1.495 (4)	C12—H12	0.9300
C2—H2A	0.9700	C13—C14	1.358 (4)
C2—H2B	0.9700	C14—H14	0.9300
C3—C4	1.382 (4)		
			
O1^i^—Cu1—N2	168.33 (9)	C5—C4—C3	118.9 (4)
O1^i^—Cu1—N1	93.65 (9)	C5—C4—H4	120.5
N2—Cu1—N1	86.74 (9)	C3—C4—H4	120.5
O1^i^—Cu1—O1	78.44 (8)	C6—C5—C4	119.9 (3)
N2—Cu1—O1	91.22 (8)	C6—C5—H5	120.0
N1—Cu1—O1	119.75 (9)	C4—C5—H5	120.0
O1^i^—Cu1—Cl2	94.50 (6)	C5—C6—C7	119.1 (3)
N2—Cu1—Cl2	93.82 (7)	C5—C6—H6	120.4
N1—Cu1—Cl2	132.43 (7)	C7—C6—H6	120.4
O1—Cu1—Cl2	107.80 (6)	N1—C7—C6	121.3 (3)
C3—N1—C7	119.7 (3)	N1—C7—H7	119.3
C3—N1—Cu1	117.75 (19)	C6—C7—H7	119.3
C7—N1—Cu1	122.2 (2)	N2—C8—C9	128.2 (3)
C8—N2—C1	117.5 (2)	N2—C8—H8	115.9
C8—N2—Cu1	125.7 (2)	C9—C8—H8	115.9
C1—N2—Cu1	116.79 (18)	C14—C9—C10	118.9 (3)
C10—O1—Cu1^i^	129.79 (18)	C14—C9—C8	115.7 (3)
C10—O1—Cu1	128.60 (17)	C10—C9—C8	125.4 (2)
Cu1^i^—O1—Cu1	101.56 (8)	O1—C10—C11	120.9 (3)
N2—C1—C2	111.4 (2)	O1—C10—C9	120.7 (3)
N2—C1—H1A	109.3	C11—C10—C9	118.4 (3)
C2—C1—H1A	109.3	C12—C11—C10	121.6 (3)
N2—C1—H1B	109.3	C12—C11—H11	119.2
C2—C1—H1B	109.3	C10—C11—H11	119.2
H1A—C1—H1B	108.0	C13—C12—C11	119.2 (3)
C3—C2—C1	113.7 (3)	C13—C12—H12	120.4
C3—C2—H2A	108.8	C11—C12—H12	120.4
C1—C2—H2A	108.8	C14—C13—C12	121.3 (3)
C3—C2—H2B	108.8	C14—C13—Cl1	119.0 (2)
C1—C2—H2B	108.8	C12—C13—Cl1	119.6 (3)
H2A—C2—H2B	107.7	C13—C14—C9	120.5 (3)
N1—C3—C4	121.0 (3)	C13—C14—H14	119.7
N1—C3—C2	115.7 (3)	C9—C14—H14	119.7
C4—C3—C2	123.3 (3)		
			
O1^i^—Cu1—N1—C3	−146.8 (2)	C1—C2—C3—N1	−66.8 (3)
N2—Cu1—N1—C3	44.9 (2)	C1—C2—C3—C4	112.9 (3)
O1—Cu1—N1—C3	134.4 (2)	N1—C3—C4—C5	−0.2 (5)
Cl2—Cu1—N1—C3	−47.3 (2)	C2—C3—C4—C5	−179.8 (3)
O1^i^—Cu1—N1—C7	40.0 (2)	C3—C4—C5—C6	−0.3 (6)
N2—Cu1—N1—C7	−128.3 (2)	C4—C5—C6—C7	0.7 (6)
O1—Cu1—N1—C7	−38.7 (2)	C3—N1—C7—C6	0.2 (4)
Cl2—Cu1—N1—C7	139.5 (2)	Cu1—N1—C7—C6	173.2 (2)
O1^i^—Cu1—N2—C8	28.7 (6)	C5—C6—C7—N1	−0.7 (5)
N1—Cu1—N2—C8	121.0 (2)	C1—N2—C8—C9	−179.8 (3)
O1—Cu1—N2—C8	1.2 (2)	Cu1—N2—C8—C9	1.8 (4)
Cl2—Cu1—N2—C8	−106.7 (2)	N2—C8—C9—C14	177.1 (3)
O1^i^—Cu1—N2—C1	−149.7 (4)	N2—C8—C9—C10	−4.4 (5)
N1—Cu1—N2—C1	−57.5 (2)	Cu1^i^—O1—C10—C11	4.6 (4)
O1—Cu1—N2—C1	−177.2 (2)	Cu1—O1—C10—C11	−178.7 (2)
Cl2—Cu1—N2—C1	74.8 (2)	Cu1^i^—O1—C10—C9	−175.26 (18)
O1^i^—Cu1—O1—C10	−177.4 (3)	Cu1—O1—C10—C9	1.4 (4)
N2—Cu1—O1—C10	−2.9 (2)	C14—C9—C10—O1	−179.1 (3)
N1—Cu1—O1—C10	−89.8 (2)	C8—C9—C10—O1	2.5 (4)
Cl2—Cu1—O1—C10	91.5 (2)	C14—C9—C10—C11	1.1 (4)
O1^i^—Cu1—O1—Cu1^i^	0.0	C8—C9—C10—C11	−177.4 (3)
N2—Cu1—O1—Cu1^i^	174.53 (10)	O1—C10—C11—C12	177.3 (3)
N1—Cu1—O1—Cu1^i^	87.59 (11)	C9—C10—C11—C12	−2.9 (4)
Cl2—Cu1—O1—Cu1^i^	−91.06 (8)	C10—C11—C12—C13	1.8 (5)
C8—N2—C1—C2	−159.3 (3)	C11—C12—C13—C14	1.1 (5)
Cu1—N2—C1—C2	19.3 (3)	C11—C12—C13—Cl1	−177.7 (2)
N2—C1—C2—C3	50.8 (4)	C12—C13—C14—C9	−2.9 (5)
C7—N1—C3—C4	0.2 (4)	Cl1—C13—C14—C9	175.9 (2)
Cu1—N1—C3—C4	−173.1 (2)	C10—C9—C14—C13	1.7 (4)
C7—N1—C3—C2	179.8 (3)	C8—C9—C14—C13	−179.7 (3)
Cu1—N1—C3—C2	6.5 (3)		

Symmetry codes: (i) −*x*+1, −*y*+2, −*z*.

## References

[bb2] Du, M., Guo, Y.-M., Chen, S.-T., Bu, X.-H. & Ribas, J. (2003). *Inorg. Chim. Acta*, **346**, 207–214.

[bb3] Rojas, D., García, A. M., Vega, A., Moreno, Y., Venegas-Yazigi, D., Garland, M. T. & Manzur, J. (2004). *Inorg. Chem.* **43**, 6324–6330. 10.1021/ic049648t 15446879

[bb4] Sheldrick, G. M. (1996). *SADABS* University of Göttingen, Germany.

[bb5] Sheldrick, G. M. (2008). *Acta Cryst.* A**64**, 112–122. 10.1107/S0108767307043930 18156677

[bb6] Siemens (1996). *SMART* and *SAINT* Siemens Analytical X-ray Instruments Inc., Madison, Wisconsin, USA.

[bb7] Yamada, S. (1999). *Coord. Chem. Rev.* **190–192**, 537–555.

